# Whole-genome sequencing of major malaria vectors reveals the evolution of new insecticide resistance variants in a longitudinal study in Burkina Faso

**DOI:** 10.1186/s12936-024-05106-7

**Published:** 2024-09-17

**Authors:** Mahamadi Kientega, Chris S. Clarkson, Nouhoun Traoré, Tin-Yu J. Hui, Samantha O’Loughlin, Abdoul-Azize Millogo, Patric Stephane Epopa, Franck A. Yao, Adrien M. G. Belem, Jon Brenas, Alistair Miles, Austin Burt, Abdoulaye Diabaté

**Affiliations:** 1 Institut de Recherche en Sciences de la Santé (IRSS), 01 BP 545 Bobo-Dioulasso 01, Burkina Faso; 2 Vector Surveillance Programme, Genomic Surveillance Unit, Wellcome Sanger Institute, Hinxton, Cambridge, UK; 3 Université Nazi Boni, 01 BP 1091 Bobo-Dioulasso, Burkina Faso; 4 Department of Life Sciences, Imperial College London, Silwood Park, Ascot, SL5 7PY UK; 5 Institut des Sciences des Sociétés, 03 BP 7047 Ouagadougou 03, Burkina Faso

**Keywords:** Insecticide resistance, Genomic surveillance, *An. gambiae*, Malaria

## Abstract

**Background:**

Intensive deployment of insecticide based malaria vector control tools resulted in the rapid evolution of phenotypes resistant to these chemicals. Understanding this process at the genomic level is important for the deployment of successful vector control interventions. Therefore, longitudinal sampling followed by whole genome sequencing (WGS) is necessary to understand how these evolutionary processes evolve over time. This study investigated the change in genetic structure and the evolution of the insecticide resistance variants in natural populations of *Anopheles gambiae* over time and space from 2012 to 2017 in Burkina Faso.

**Methods:**

New genomic data have been generated from *An. gambiae* mosquitoes collected from three villages in the western part of Burkina Faso between 2012 and 2017. The samples were whole-genome sequenced and the data used in the *An. gambiae* 1000 genomes (Ag1000G) project as part of the Vector Observatory. Genomic data were analysed using the analysis pipeline previously designed by the Ag1000G project.

**Results:**

The results showed similar and consistent nucleotide diversity and negative Tajima’s D between *An. gambiae *sensu stricto (s.s.) and *Anopheles coluzzii*. Principal component analysis (PCA) and the fixation index (*F*_*ST*_) showed a clear genetic structure in the *An. gambiae *sensu lato (s.l.) species. Genome-wide *F*_*ST*_ and H12 scans identified genomic regions under divergent selection that may have implications in the adaptation to ecological changes. Novel voltage-gated sodium channel pyrethroid resistance target-site alleles (*V402L, I1527T*) were identified at increasing frequencies alongside the established alleles (*Vgsc-L995F*, *Vgsc-L995S* and *N1570Y*) within the *An. gambiae *s.l. populations. Organophosphate metabolic resistance markers were also identified, at increasing frequencies, within the *An. gambiae *s.s. populations from 2012 to 2017, including the SNP *Ace1-G280S* and its associated duplication. Variants simultaneously identified in the same vector populations raise concerns about the long-term efficacy of new generation bed nets and the recently organophosphate pirimiphos-methyl indoor residual spraying in Burkina Faso.

**Conclusion:**

These findings highlighted the benefit of genomic surveillance of malaria vectors for the detection of new insecticide resistance variants, the monitoring of the existing resistance variants, and also to get insights into the evolutionary processes driving insecticide resistance.

**Supplementary Information:**

The online version contains supplementary material available at 10.1186/s12936-024-05106-7.

## Background

Malaria remains a major public health problem in Africa [1]. *Anopheles gambiae *sensu lato (s.l.), the main malaria vector in Africa, exhibits high phenotypic and genotypic diversity, allowing rapid adaptation to environmental changes, such as the introduction of new insecticidal control methods [2]. In Burkina Faso, the major malaria vectors *An. gambiae *sensu stricto (s.s.), *Anopheles coluzzii* and *Anopheles arabiensis*, members of the *An. gambiae* complex, can be found living in sympatry, with divergent resting and feeding behaviours [3, 4]. These species, through their ability to colonize different ecological settings, their preference for human blood feeding, and their high susceptibility to a malaria parasite (*Plasmodium falciparum*) infection, are responsible for around 90% of the malaria burden in Burkina Faso and the whole sub-Saharan Africa [5, 6].

The National Malaria Control Programme (NMCP) of Burkina Faso and their partners have invested a lot to increase the coverage of long-lasting insecticidal bed nets (LLINs) and other control tools such as indoor residual spraying (IRS) of insecticides to drop the malaria transmission line in the country. The coverage of LLINs in households increased significantly in the country over the sampling periods, from 5.6% in 2003 to about 80% in 2014, reaching 83% in 2021 [7, 8]. The recent 2019 and 2022 bed net campaigns involved the free distribution of three types of LLINs, especially the piperonyl butoxide (PBO)-synergist, the dual-AI Interceptor G2 (containing the pyrrole insecticide, chlorfenapyr) and the standard LLINs [9]. Despite these achievements, malaria remains a countrywide health problem responsible in 2021 for about 12.2 million cases and 4355 deaths [10].

In Burkina Faso and most sub-Saharan countries, the primary vector surveillance strategies relied on the routine tracking of the vector species density and distribution and the monitoring of phenotypic insecticide resistance (IR) status. Unfortunately, these methods proved unable to adequately explain the genetic and evolutionary processes driving the increasing spread of IR variants and changes in vector behaviour [11]. Recent advances in genomic sequencing technologies, both price and throughput, and new cloud native analysis tools [12] have made genomic surveillance of malaria vectors possible. Genomic surveillance has advantages to provide insights on the genetic and evolutionary phenomena of vectors that could have impact on vector control strategies. Towards this aim, the *Anopheles gambiae* 1000 genomes (Ag1000G) project driven by the MalariaGEN Vector Observatory sequenced more than 15,000 *Anopheles* mosquitoes collected in 25 African countries [13, 14]. A major goal of this project was to support malaria elimination efforts by providing a high quality open access data resource on natural genetic variation within *An. gambiae *s.s., *An. coluzzii* and *An. arabiensis* populations, both for the public health and research communities [15].

Previous studies investigating IR using these genomic data have highlighted associated genetic variants distributed throughout the mosquito genome [16–19]. The phenotypic effect of many of these variants has been demonstrated through *in-vivo* and *in-vitro* experiments and their effects are evident in the low mortality rate observed during the IR bioassays, and in the reduction of the efficacy of the insecticide-based control tools [20–22]. The strength of these resistance phenotypes and the wide geographical distribution of these variants raise concerns about the durability of the new insecticide-based vector control tools being used to overcome resistance, including indoor residual spraying (IRS) with organophosphate insecticides, such as Actellic (pirimiphos-methyl) and pyrethroid bed-nets treated with metabolic resistance blocking piperonyl butoxide (PBO) synergist.

There is an increasingly urgent need for sustainable strategies and effective tools to support malaria elimination efforts in the face of ever evolving vectors. Research of alternative and innovative tools such as genetic modified mosquitoes, new insecticidal compounds as well as natural symbionts has increased in recent years, giving a glimmer of hope to people living in endemic areas [23, 24]. However, given the rapid evolution of the of the malaria vector genome, as demonstrated by many studies [13, 25, 26], any implementation of vector control tools requires a better understanding of the genomic variation of vector species involved, in particular an understanding of the evolutionary processes, emergence and spread of IR-associated variants in natural populations over time. Thus, longitudinal sampling of vectors, followed by whole-genome population genomics becomes a very helpful tool upstream and downstream of the implementation of the control tools to provide insights on the genetic and evolutionary phenomena of vectors that could have any impact on vector control strategies. This study used the genomic data from 1409 *An. gambiae *s.l. samples, collected in Burkina Faso between 2012 and 2017 and sequenced as part of the *Anopheles gambiae* 1000 Genomes Project (2017, 2020) to characterize the spread of known IR variants over space and time, while analysing these natural populations for evidence of the evolution of novel IR mechanisms that could threaten current or future vector control methods.

## Methods

### Mosquitoes sampling

**Fig. 1:**
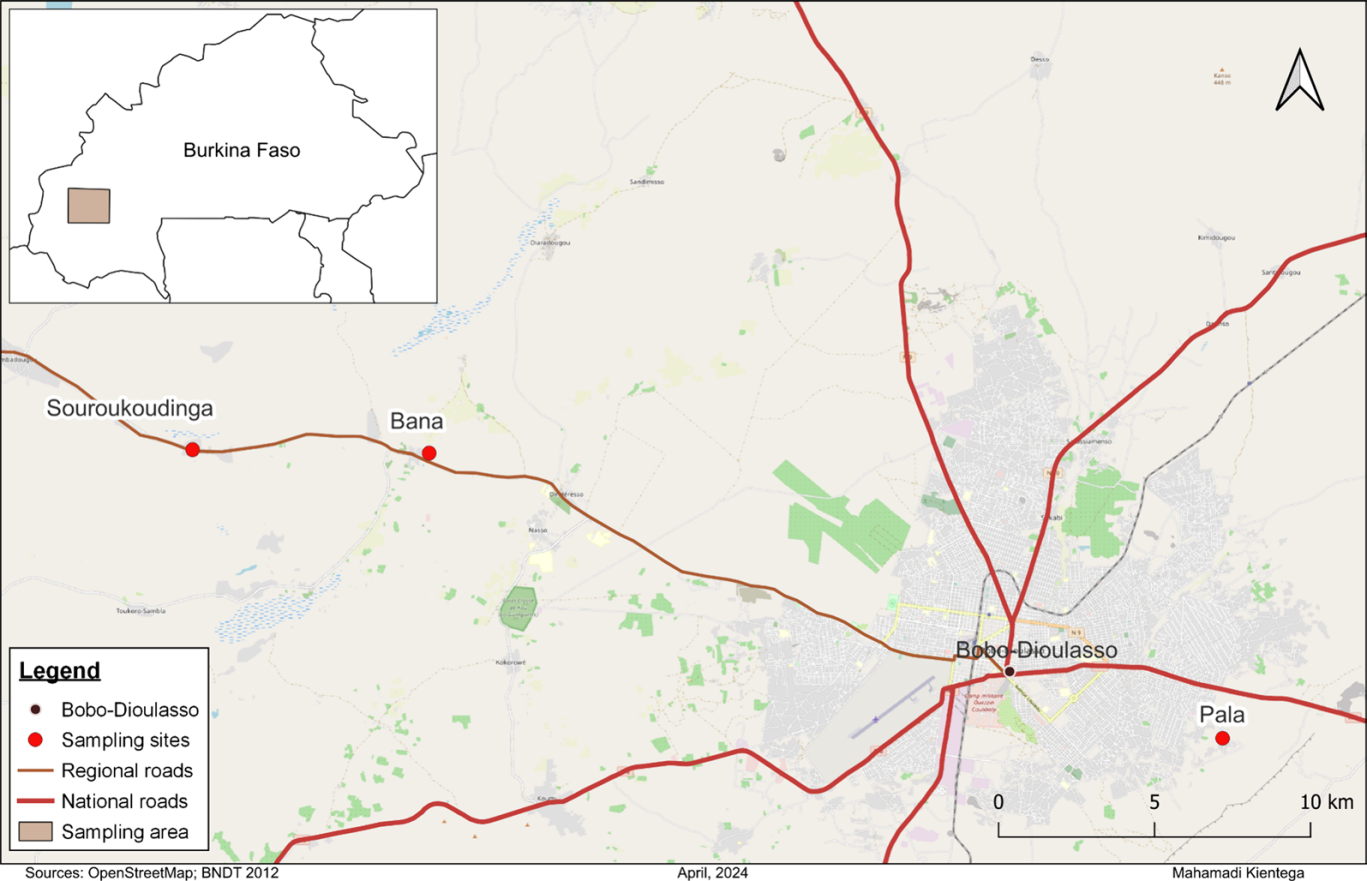
Map of the sampling sites around Bobo-Dioulasso city

Mosquito samples were collected by the Target Malaria project in three villages, Bana (11.233, − 4.472), Souroukoudinga (11.235, − 4.535) and Pala (11.150, − 4.235), located in the western part of Burkina Faso (Fig. 1) during the rainy season from 2012 to 2017. These villages have very similar ecological and climatic conditions, i.e. a humid savannah zone surrounding the Bobo-Dioulasso city and are characterized by two seasons: a rainy period from June to October and a dry season from November to April. The average annual rainfall is above 800 mm, with an average annual temperature and humidity of about ~ 27 °C and ~ 60%, respectively [27]. The main agricultural practices are cotton and cereal (incl. maize, rice, sorghum) cultivation utilizing large amount of pesticides [28].

Malaria remains endemic in the area surrounding the Bobo-Dioulasso city, causing 338 deaths in the three health districts of the city [10]. The *Anopheles gambiae* complex species remain the major malaria vector in the sampling area [27]. Malaria control relies on the anti-malarial drugs and prevention of mosquito bites using long-lasting insecticidal nets (LLINs), the most widespread vector control tool, and to a lesser extent indoor residual spraying (IRS), sprays and mosquito-repellent coils.

Mosquitoes were collected during the rainy from 2012 to 2017 using three collection methods: Pyrethroid spray catches (PSC), human landing catches (HLC) and swarm collection [29]. PSC and HLC were used for female mosquito collection, whereas males were collected in swarms. After collection, mosquito samples were morphologically identified using the morphological keys [30] and *An. gambiae *s.l. specimens were stored in 80% ethanol and then shipped for the whole genome sequencing at the Wellcome Sanger Institute.

### Whole genome sequencing and genomic data management

*Anopheles gambiae *s.l. mosquitoes were sent for individual whole genome sequencing at high coverage (30 ×) using Illumina technology at the Wellcome Sanger Institute as part of the vector observatory. Genomic data were analysed at the MalariaGEN Resource Centre following the previous data analyses and quality clean-up pipelines designed by the Ag1000G project. Briefly, the analyses consisted of the raw data cleaning, the quality control and the mapping to the AgamP4 reference genome using BWA version 0.7.15. Indel realignment was performed using GATK version 3.7-0 RealignerTargetCreator and IndelRealigner. Single nucleotide polymorphisms were called using GATK version 3.7-0 UnifiedGenotyper. Genotypes were called for each sample independently, in genotyping mode, given all possible alleles at all genomic sites where the reference base was not “N”. Complete specifications of the alignment and genotyping pipelines are available from the malariagen/pipelines GitHub repository [31]. After variant calling, the raw sequences in FASTQ format and the aligned sequences in BAM format were stored in the European Nucleotide Archive (ENA, Study Accession n° PRJEB42254). The genetic variants in VCF and zarr formats, including the samples’ metadata, were stored on Google Cloud and are accessible via the malariagen_data package [32] or are directly downloadable. Full details about the sequencing technology, the raw data clean-up and quality control, the SNPs calling, haplotypes phasing and copy number variants identification including the storage of the genomic data are available in the MalariaGEN homepage [31].

### Population genomic and insecticide resistance variant analyses

Genetic diversity and population structure were analyzed using Jupyter Notebook in the Google Colab platform. The genetic variants (SNPs, haplotypes and CNVs) and the reference genome of *An. gambiae *s.l. were accessed using the malariagen_data package for direct analyses of the genomic data in the cloud. This package provides a wide range of functions and properties for cloud data access and for exploratory analysis of large scale genetic variation data [32]. Additional packages such as scikit-allel [33] and other python standard data-management packages were used for the data analyses and the plotting. The number of segregating sites (number of SNPs) was calculated in the whole genome (X, 2RL, 3RL) of *An. gambiae *s.l. The diversity statistics [nucleotide diversity (*θ*_*π*_), Tajima’s D (D), Watterson theta (*θ*_*w*_), allele frequency spectrum (sfs)] were calculated using the SNPs called in the 3L chromosome in all the mosquito populations. Tukey multiple comparison test was applied to compare the diversity statistic between vector populations over the sampling periods.

Genome wide selection scan was performed using the Garud H12 [34] statistic to detect the genomic region under positive selection. H12 is a haplotype statistic that combines the first and second most frequent haplotype frequencies in a sample into a single combined haplotype frequency and then recalculates haplotype homozygosity using this revised haplotype frequency distribution [34]. *F*_*ST*_ and PCA analyses were performed in the 3L chromosome to investigate the genetic structure and the differentiation of *An. gambiae *s.l. populations. The 3L chromosomal arm was used for the genetic diversity and population structure analyses because this region has no polymorphic inversions [35]. The extent and the spread of insecticide resistance variants were also investigated in the *An. gambiae *s.l. populations using the malariagen_data python package. Time series (from 2012 to 2017) SNPs frequencies were analysed in known target-site resistance associated genes, *VGSC* and *ACE1*. The copy number variation (CNV) frequencies were also investigated in genomic regions associated with metabolic resistance, cytochrome P450s, glutathione-s-transferases, acetylcholinesterase, diacylglycerol kinase and carboxylesterases.

## Results

### Population sampling

*Anopheles gambiae* mosquitoes collected in Burkina Faso from 2012 to 2017 were sequenced as part of the follow-up project, the MalariaGEN Vector Observatory*.* In total, 1409 *An. gambiae *s.l. mosquitoes (978 females and 431 males) were collected from three villages (Bana, Souroukoudinga, Pala) surrounding the Bobo-Dioulasso city in the west of Burkina Faso and shipped for deep whole-genome sequenced mosquitoes. The longitudinal mosquito sampling survey was carried out in the three villages during the rainy seasons of the years 2012, 2014, 2015, 2016 and 2017 (Table 1). The species composition of the sample set varied between the sampling sites and the time-points (Table 1). *Anopheles coluz*z*ii* was the most predominant species found, accounting for 52.73% [743/1409] of samples, followed by *An. gambiae *s.s. with 38.96% (549/1409), while *An. arabiensis* only comprised 8.16% (115/1409) of samples collected. Two *An. gambiae *s.s. and *An. coluzzii* hybrids were also identified. As shown by a previous work, *An. coluzzii* remains the most predominant species in Bana (83.87% [489/583]) and Souroukoudinga (60.90% [243/399]) while *An. gambiae *s.s. is prevalent in Pala (70.72% [302/427]) [27].

**Table 1:** Species composition of the *An. gambiae* complex of the samples collected in the three villages surrounding the city of Bobo-Dioulasso during the sampling period (2012–2017)

	*An. arabiensis*		*An. coluzzii*		*An. gambiae s.s*		*gambiae_coluzzii*	
	F	M	F	M	F	M	F	M
Bana	1 [0.17%]	0	379 [65.01%]	110 [18.87%]	29 [4.97%]	63 [10.81%]	0	1 [0.17%]
2012	0	0	38 [58.46%]	4 [6.15%]	5 [7.69%]	17 [26.15%]	0	1 [1.54%]
2014	1 [0.78%]	0	63 [48.84%]	33 [25.58%]	13 [10.08%]	19 [14.73%]	0	0
2015	0	0	95 [78.51%]	1 [0.83%]	8 [6.61%]	17 [14.05%]	0	0
2016	0	0	96 [72.18%]	24 [18.05%]	3 [2.26%]	10 [7.52%]	0	0
2017	0	0	87 [64.44%]	48 [35.56%]	0	0	0	0
Pala	78 [18.27%]	35 [8.2%]	11 [2.58%]	0	236 [55.27%]	66 [15.46%]	1 [0.23%]	0
2012	0	0	11 [18.64%]	0	40 [67.8%]	8 [13.56%]	0	0
2014	6 [6.0%]	5 [5.0%]	0	0	68 [68.0%]	21 [21.0%]	0	0
2015	58 [35.37%]	30 [18.29%]	0	0	61 [37.2%]	15 [9.15%]	0	0
2016	14 [20.90%]	0	0	0	31 [46.27%]	22 [32.84%]	0	0
2017	0	0	0	0	36 [97.3%]	0	1 [2.7%]	0
Souroukoudinga	1 [0.25%]	0	141 [35.34%]	102 [25.56%]	101 [25.31%]	54 [13.53%]	0	0
2012	0	0	29 [50.88%]	0	28 [49.12%]	0	0	0
2014	0	0	44 [44.44%]	6 [6.06%]	34 [34.34%]	15 [15.15%]	0	0
2015	0	0	35 [27.56%]	52 [40.94%]	24 [18.9%]	16 [12.6%]	0	0
2016	0	0	30 [34.88%]	18 [20.93%]	15 [17.44%]	23 [26.74%]	0	0
2017	1 [3.33%]	0	3 [10.0%]	26 [86.67%]	0	0	0	0
Total	80 [5.68%]	35 [2.48%]	531 [37.69%]	212 [15.05%]	366 [25.98%]	183 [12.99%]	1 [0.07%]	1 [0.07%]

*gambiae_coluzzii*: hybrid of *An. gambiae* s.s. and *An. coluzzii*; F: female; M: male

### Genetic diversity

**Fig. 2:**
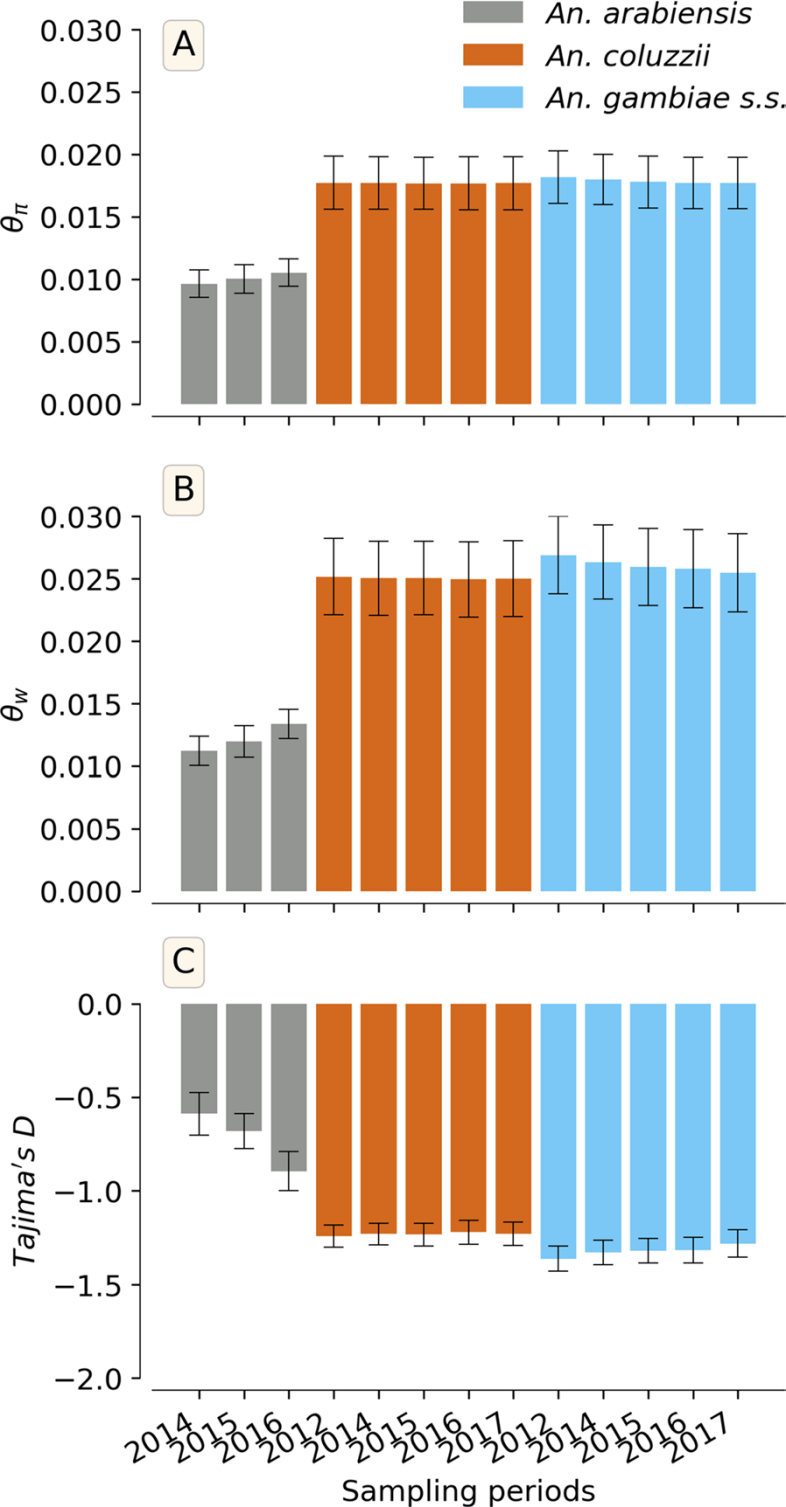
Average of genetic diversity statistics within the *An. gambiae* s.l. populations in the study sites from 2012 to 2017; The Y-axis shows the diversity statistic, the X-axis shows the sampling periods and the colours show the populations. **A** Nucleotide diversity (*θ*_*π*_), **B** Watterson theta (*θ*_*w*_), **C** Tajima’s D, error bars correspond to the 95% confidence interval for each year

Understanding genetic diversity allows us to compare demography of species over time and space. Analyses of these data showed around 27.94 million of segregating sites [~ 91% of biallelic SNPs (Single Nucleotide Polymorphism)] in the *An. gambiae *s.l. genome. Approximately 25.68 million of SNPs were identified in the autosomes and 2.262 million of SNPs in the X chromosome. Analyses to assess the genetic diversity (see Methods section) showed almost similar nucleotide diversity (*θ*_*π*_), Tajima’s D and Watterson’s theta (*θ*_*w*)_ over all the sampling periods in *An. gambiae* and *An. coluzzii*, as in most natural populations [36] across these species range (Fig. 2), possibly indicating similar demographic histories between these two species. In contrast, *An. arabiensis* showed lower diversity, though with apparent increase over the sampling periods. The diversity findings were reflected in a genome-wide negative Tajima’s D, caused by an excess of rare variants due to either positive selection and/or rapid demographic changes through population expansion. In addition, the allelic frequency spectrum (Fig. S1) also showed the same trend highlighting the excess of low frequency variants in the *An. gambiae *s.l. genome.

### Population structure

**Fig. 3:**
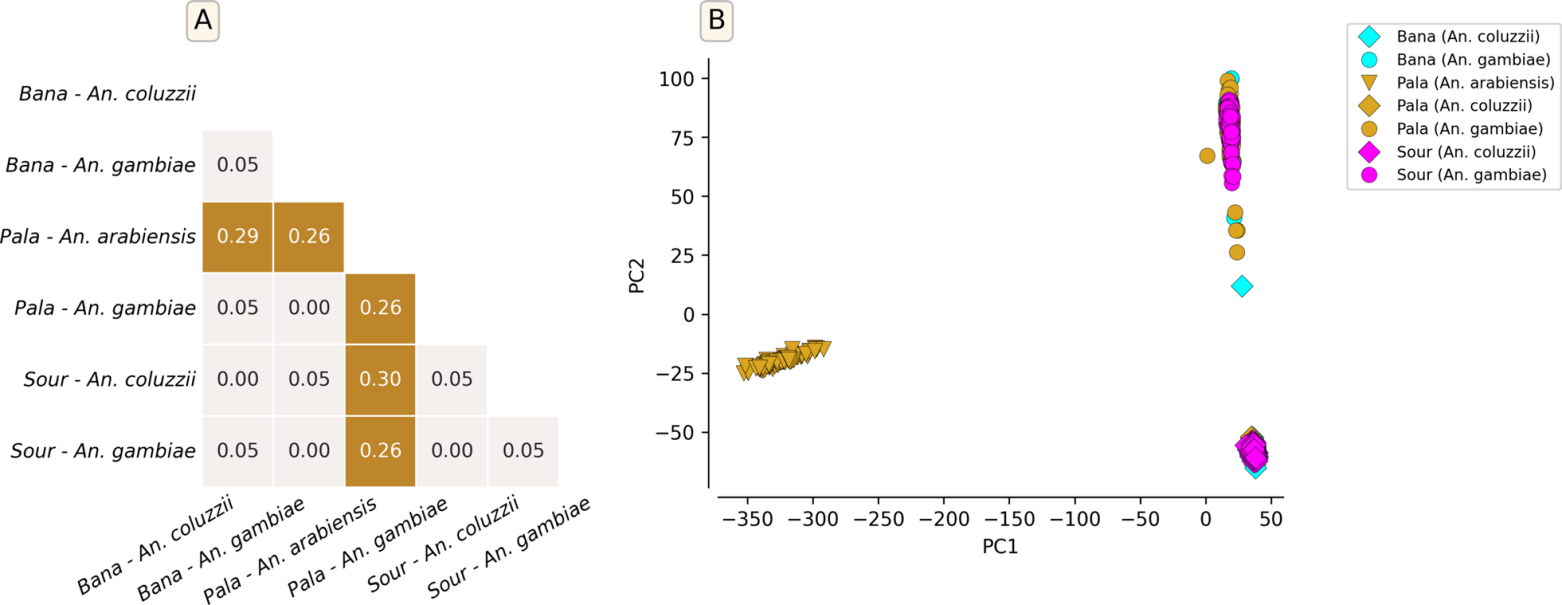
Genetic differentiation and population structure of *An. gambiae* s.l. using the SNPs identified in the 3L chromosome. Sour: Souroukoudinga; **A** Pairwise *F*_*ST*_ between *An. gambiae* s.l. populations collected in different villages; no differentiation between populations of the same species collected in two different villages; **B** PCA showing the genetic structure of *An. gambiae* s.l*.* populations

Understanding population structure between, and within, vector species is important not only because of its implications for gene flow of medically relevant alleles or the role of gene drive control methods, but also because hidden structure will confound downstream genomic analyses of the population. Consequently, the genetic structure and the patterns of gene flow were investigated using SNPs on the 3L chromosome (see Methods section). Pairwise *F*_*ST*_ was estimated between and within species collected in the different villages and was found to be low (0 to 0.05) between *An. gambiae* and *An. coluzzii* (Fig. 3A). These results support other evidence for ongoing gene flow between *An. coluzzii* and *An. gambiae *s.s. [37]. However, for *An. arabiensis*, results showed relatively higher *F*_*ST*_ between both *An. coluzzii* and *An. gambiae*, ranging from 0.26 to 0.30 (Fig. 3A). No differentiation (*F*_*ST*_ ~ 0) was observed between populations of the same species collected in different villages, suggesting no population structure on this scale.

The principal component analysis (PCA) showed a clear divergence between the *An. gambiae s.l.* species. Examining the first two principal components, samples fell into three clusters, where individuals of the same species showed higher genetic similarities among themselves compared to individuals of other species. No sub-clusters were observed within each species (Fig. 3B). No change was observed in the population structure of each species throughout the sampling periods, and the sampling sites (Fig. S2); however, some other genomic regions were shown to be under divergent selection (see Organophosphate and carbamate resistance: increasing threats to control plus a potential novel IR mechanism, Fig. S3). The lack of genetic structure (low *F*_*ST*_ ~ *0.05*) between *An. coluzzii* and *An. gambiae *s.s. in the sampling area suggests showed that these species may share advantageous allelic variants, such as those involved in insecticide resistance.

### Insecticide resistance

#### Pyrethroid target-site resistance: concerning increase of new mutations

**Fig. 4:**
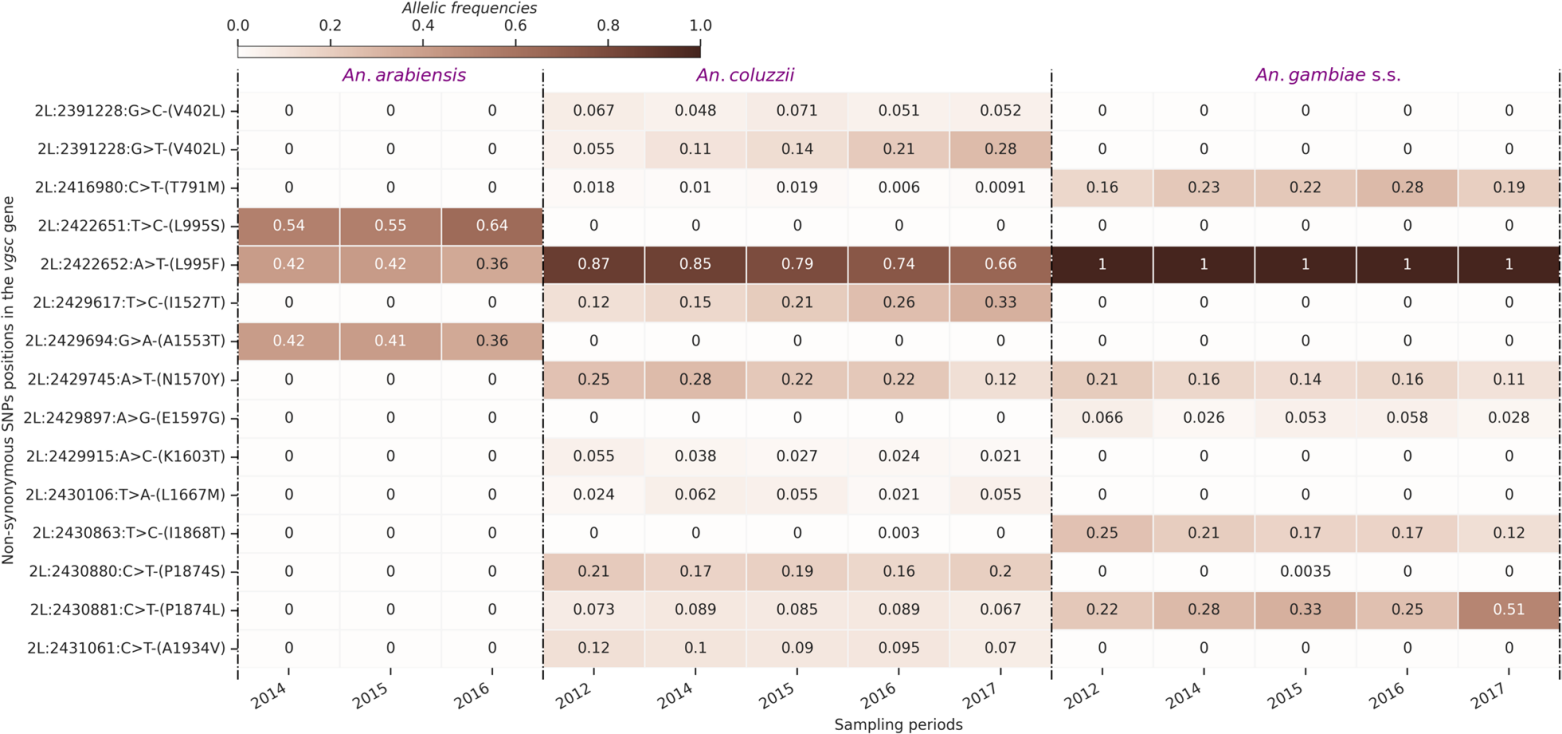
Heat map showing the frequencies of the non-synonymous SNPs frequencies (max freq > 0.05 in at least one population) in the *VGSC* gene in the *An. gambiae *s.l. populations over time. The X-axis shows the *An. gambiae *s.l. populations and the sampling periods. The Y-axis shows the non-synonymous SNPs positions in the chromosome 2L and the corresponding amino acid change. The gradient colour bar shows the distribution of the allelic frequencies

Pyrethroid target-site resistance in *An. gambiae *s.l. has, historically, been primarily driven by two main “*kdr (knock down resistance)”* SNPs, causing the amino acid changes *vgsc-L995F* and *vgsc-L995S* [18]. These mutations are widespread and are present at high frequencies among populations in many regions in Africa [13]. Improvements in sequencing technologies have allowed the identification of many additional SNPs in the voltage gated sodium channel gene *(VGSC [2L: 2358158–2431617])* gene that could enhance the level of pyrethroid resistance over time [39, 40]. This study identified 634 non-synonymous SNPs within the *VGSC* gene in all the *An. gambiae *s.l. populations (Table S1). The *kdr* allele *vgsc-L995F* was identified at high frequencies (> 30%) in all the populations, including *An. arabiensis*, and was found at fixation in *An. gambiae *s.s. collections since 2012 (Fig. 4). The N1570Y allele, previously shown to increase the level of pyrethroid resistance when in concert with *vgsc-L995F* [40], was also present in both *An. gambiae *s.s. and *An. coluzzii* populations at around 30% frequency, but it was not found in *An. arabiensis.*

**Fig. 5:**
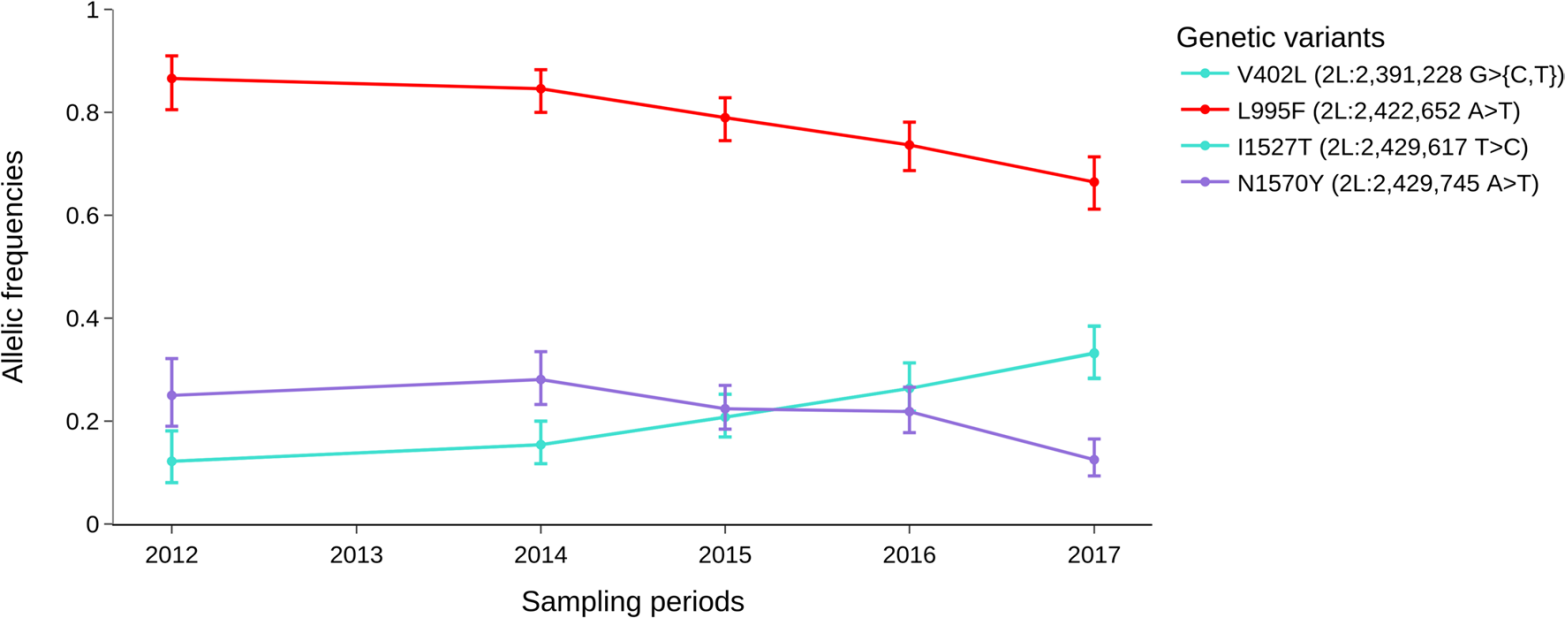
Dynamics of *vgsc-L995F*, *vgsc-V402L* + *I1527T* and N1570Y allele frequencies in the *An. coluzzii* populations; *vgsc-V402L* and *vgsc-I1527T* are shown to be linked and exhibited the same frequencies from 2012 to 2017, the X-axis shows the sampling periods and the Y-axis shows the allelic frequencies

A *VGSC* haplotype carrying the apparently linked amino acid substitutions *vgsc-V402L* + *I1527T* was also identified in Burkina Faso *An. coluzzii* (Figs. 4 and 5). Two alleles of the *vgsc-V402L* were detected, the *2L: 2 391 228 G* > *C (V402L)* with a relatively constant frequency ranging from 6.7% in 2012 to 5.2% in 2017 and the *2L: 2 391 228 G* > *T (V402L)* with an evolving frequency from 5.5% in 2012 to 28% in 2017. However, the *vsgc- I1527T* is monoallelic and identified with an evolving frequency 12% in 2012 to 33% in 2017 (Fig. 4). A recent in-vivo investigation has shown that the *vgsc-V402L* substitution results in a pyrethroid resistance phenotype with lower fitness costs than the classic *vgsc-L995F kdr* mutation [39]. Upon discovery of *vgsc-V402L* + *I1527T,* it was suggested that this haplotype may be a “relic” resistance haplotype, pre-dating and being overtaken by a more effective *vgsc-L995F* carrying haplotype [18]. However, the time series data shows that the inverse appears to be the case. The *vgsc-V402L* + *I1527T* increased from 12.19% in 2012 to 33.12% in 2017, and the sum of the frequencies of the two *vgsc-V402L* alleles (*2L: 2 391 228 G* > *{C, T}*) is approximately equal to the frequency of *vgsc-I1527T* during the sampling periods (Figs. 4 and 5). The increase of these alleles coincides with the drop in the frequency of the *vgsc-L995F* mutation in *An. coluzzii* populations from 86.58% in 2012 to 66.46% in 2017 (Figs. 4 and 5). These results and previous works [18, 39] suggest these two alleles are increasing in frequency and seem to replace the *vgsc-L995F* alleles in *An. coluzzii* populations, but had not spread to *An. gambiae *s.s. The increased emergence of new *kdr* alleles alongside the existing *kdr* mutations (*vgsc-L995F* and *vgsc-L995S*) at high frequencies may increase the resistance level of vector populations to pyrethroid compounds.

#### Pyrethroid metabolic resistance: increasing CNV frequencies.

Gene duplications and deletions (copy number variants—CNVs) are known to influence the expression level of genes by deleting or providing additional copies of the original one. Consequently, CNVs of genes involved in xenobiotic detoxification can increase insecticide metabolism or reduce the expression of genes with high fitness cost, producing a resistance phenotype [16]. To help overcome pyrethroid resistance, now commonplace in many natural populations, PBO synergist bed nets have recently been introduced. These bed nets enhance the pyrethroid efficacy by inhibiting the cytochrome P450 enzymes that can be responsible for metabolic pyrethroid resistance [41]. Often, mechanisms driving the resistance in a given area remain unclear [20], however, whole genome sequencing allows CNV calling, and the detection of resistance associated alleles [17].

**Fig. 6:**
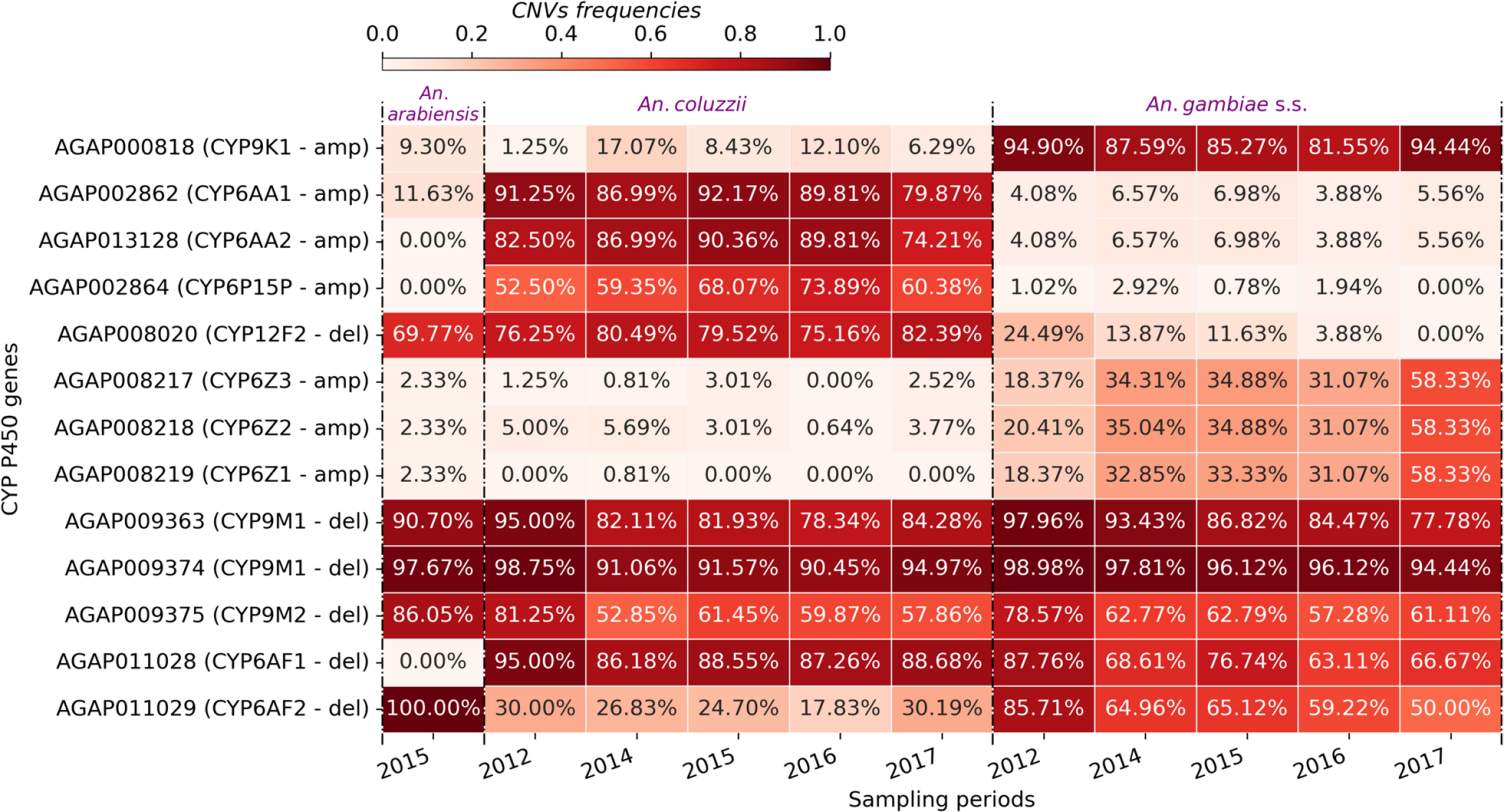
Heat map of the cytochrome P450 genes showing high CNVs frequencies in the *An. gambiae* s.l. populations. The X-axis shows the *An. gambiae* s.l. populations and the sampling periods. The Y-axis shows the positions of the P450 genes ID and the CNV type (del: deletion or amp: amplification). The gradient colour bar shows the distribution of the CNV frequencies

CNVs were identified in 136 Cytochrome P450 genes distributed across the whole genome and most of these (~ 63.97%) were gene amplifications (Table S2). The majority of these CNVs were species-specific, and some were found at increasing frequencies over time in the three species. Some genes showing low CNVs frequencies in 2012 displayed increasing CNVs frequencies in the following years (Fig. 6). This situation was observed in the *CYP6Z* cluster of genes (*CYP6Z1, CYP6Z2 and CYP6Z3*), known to be associated with pyrethroid resistance [16], whose CNVs frequencies increased from 17% in 2012 to up to 60% in 2017 in *An. gambiae *s.s. populations. Additionally, the *CYP9K1* gene, also previously shown to be involved in pyrethroid resistance, showed CNVs at high frequencies up to 94% from 2012 to 2017. In the *An. coluzzii* populations, CNVs were identified at frequencies ranging from 50% in 2012 to up to 90% in 2017 within the *CYP6AA1, CYP6AA2, CYP6P15P* and *CYP12F2* genes. CNVs related to gene deletions rate was shown to be high in the vector populations and seems to be associated with metabolic resistance. The results showed approximately 36.03% of CYP P450 genes having CNVs related to gene deletion in the vector populations. Interestingly, some of these genes such as the *CYP6AF* (*CYP6AF1* and *CYP6AF2*) and *CYP9M* cluster (*CYP9M1 and CYP9M2*) were shown to be deleted in 50–100% of the vectors populations except *An. arabiensis* for the gene *CYP6AF1* (Fig. 6). The presence of both CNVs types in the same mosquito populations can increase and/or decrease the expression level of some genes to enhance mosquito fitness response to face the environmental forces like insecticide pressure. Further studies are strongly needed to understand the impact of these new variants in the mosquito fitness and vector control tools.

### Organophosphate and carbamate resistance: increasing threats to control plus a potential novel IR mechanism

Indoor residual spraying (IRS) is one of the major vector control strategies in Africa [42]. However, the coverage of this strategy dropped considerably over the sampling periods because of the emergence of resistance to the pyrethroid compounds and also the low residual efficacy of carbamates compounds [43]. Recently, IRS with Actellic 300 CS containing pirimiphos-methyl, an organophosphate as the active ingredient, showed interesting results including a prolonged residual efficacy up to seven months and a reduction of vector density in many sprayed areas in Africa [44–46]. This new formulation demonstrated better ability to target pyrethroid resistant and indoor resting mosquitoes and also showed additional benefit when used in combination with LLINs [47, 48].

Longitudinal sampling was used to assess the extent of organophosphate and carbamate resistance in Burkina Faso that could threaten the long-term efficacy of pirimiphos-methyl-based IRS. A sliding window genome-wide *F*_*ST*_ scan showed a year to year (from 2012 to 2017) genetic difference in some regions of the genome especially on chromosomes 2 and X in *An. gambiae *s.s. and *An. coluzzii* samples. Most of these changes were observed in genomic regions previously shown to be associated with insecticide-resistance suggesting a signal of positive selection in these genes (Figs. 8 and 9).

**Fig. 7:**
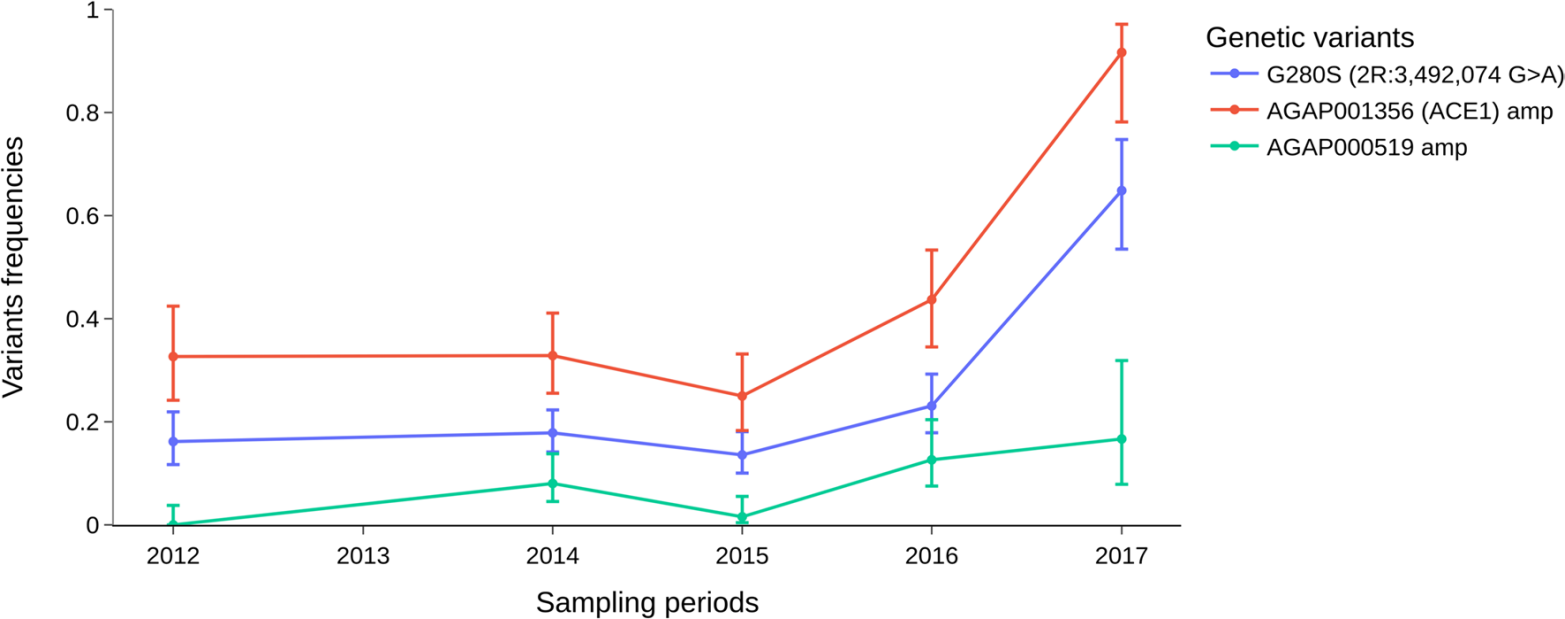
Evolution of variant frequencies of the *ACE1* (*Ace1-G280S, AGAP001356 (ACE1) amp*) and Diacylglycerol Kinase (*AGAP000519 amp*) genes in the *An. gambiae* s.s. populations. The X axis shows the sampling periods and the Y axis, the allele frequencies of the genetic variants

Both *ACE1 (2R: 3484107–3495790)* and Diacylglycerol Kinase (DGK or AGAP000519 [X: 9215504–9266532]) loci showed no selection signal in 2012 in any vector populations sampled, but by 2017 selection signals were detected at both loci in *An. gambiae s.s.* populations. The increasing gene amplification signal in the *ace1* locus, from 32.65% in 2012 to up to 91.67% in 2017, was corroborated by the increase in the insecticide resistance associated with *ace1-G280S* variant previously shown to be involved in organophosphate and carbamate resistance [49] (from 15.81% in 2012 to 65.27% in 2017), but only in *An. gambiae s.s.* populations (Fig. 7). A total of 243 other non-synonymous SNPs in the *ace1* gene (Table S3). CNV amplifications were also found in *An. coluzzii* populations, but at low and constant frequencies (~ 3%), but not in *An. arabiensis* populations.

Genetic variants of the *GSTe* genes have also been reported to be involved in resistance to organophosphates [50]. The analyses identified 41 *GSTe* genes with CNVs including 24 genes with amplifications and 17 genes with deletions. These CNVs are distributed across the genome (Table S4). Most of these CNVs were found at frequencies below 50% except the deletions of *GSTD5* in 60% to 100% of all the vector populations (Fig. S4, Table S4).

**Fig. 8:**
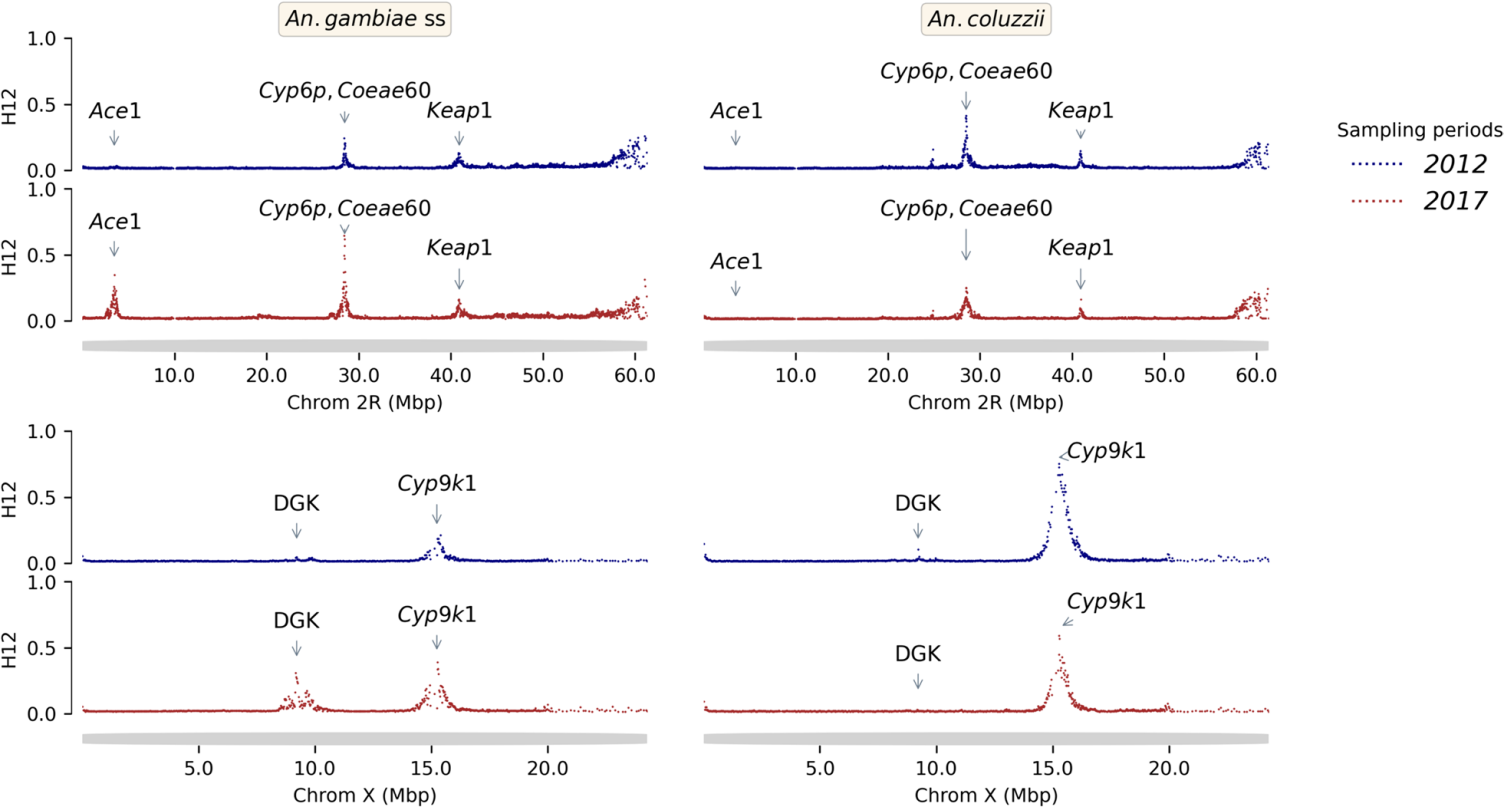
Genome-wide scan for recent selection using Garud’s H12 across chromosome X and arm 2R (5 kb window) of the *An. gambiae *s.s. and *An. coluzzii* populations from 2012 to 2017 showed positive selection signals mainly in the genomic region involved in insecticide resistance. The X-axis shows the genome (chromosome) positions and the Y-axis shows the Garud’ H12 values. The top and bottom left panels show the selection signal in *An. gambiae* s.s. populations, and the top and bottom right panels show the signal in *An. colozzii* populations. The colours represent the sampling periods, where the navy colour is 2012 and the brown colour is 2017. High values of H12 indicate a signal of positive selection in the corresponding genomic region within the populations: Ace1: Acetylcholinesterase gene; Cyp6p: Cytochrome P450 gene; Coeae60: Carboxyl-esterase gene; Keap1: Kelch-like ECH-Associated Protein 1; Dgk: Diaglycerol Kinase (AGAP000519) gene; Cyp9k1: Cytochrome P450 gene

The carboxylesterases (COEs) enzymes have been reported to be involved in the rapid metabolization of both pyrethroid and organophosphate compounds [51] and consequently could have an impact on the benefit of the combined organophosphate-based IRS and pyrethroid-based LLINs approach. In this study, CNVs were identified in 39 genes across the genome, most of these CNVs were gene amplifications, only 25.64% were deletions (Table S5). The frequencies of these CNVs were relatively low, except those of the *COEAE*60 gene which were identified at frequencies above 60% in *An. coluzzii* populations from 2012 to 2017 (Fig. S5). Additionally, H12 analysis revealed a positive selection signal in the *COEAE*60 gene (Fig. 8). The COEAE*G cluster genes (*COEAE2G, COEAE3G, COEAE5G, COEAE6G* and *COEAE7G*) and the *Coeae3H* gene showed CNVs at increasing frequencies from 11% in 2012 to up to 25% in 2017 in the *An. gambiae *s.s. populations. The *COEJHE3E* gene showed CNVs at 32.56% frequencies in *An. arabiensis*, and at frequencies higher than 30% in *An. gambiae *s.s. populations (except for sampling in 2017 where its frequency was 19.4%) (Fig. S5).

**Fig. 9:**
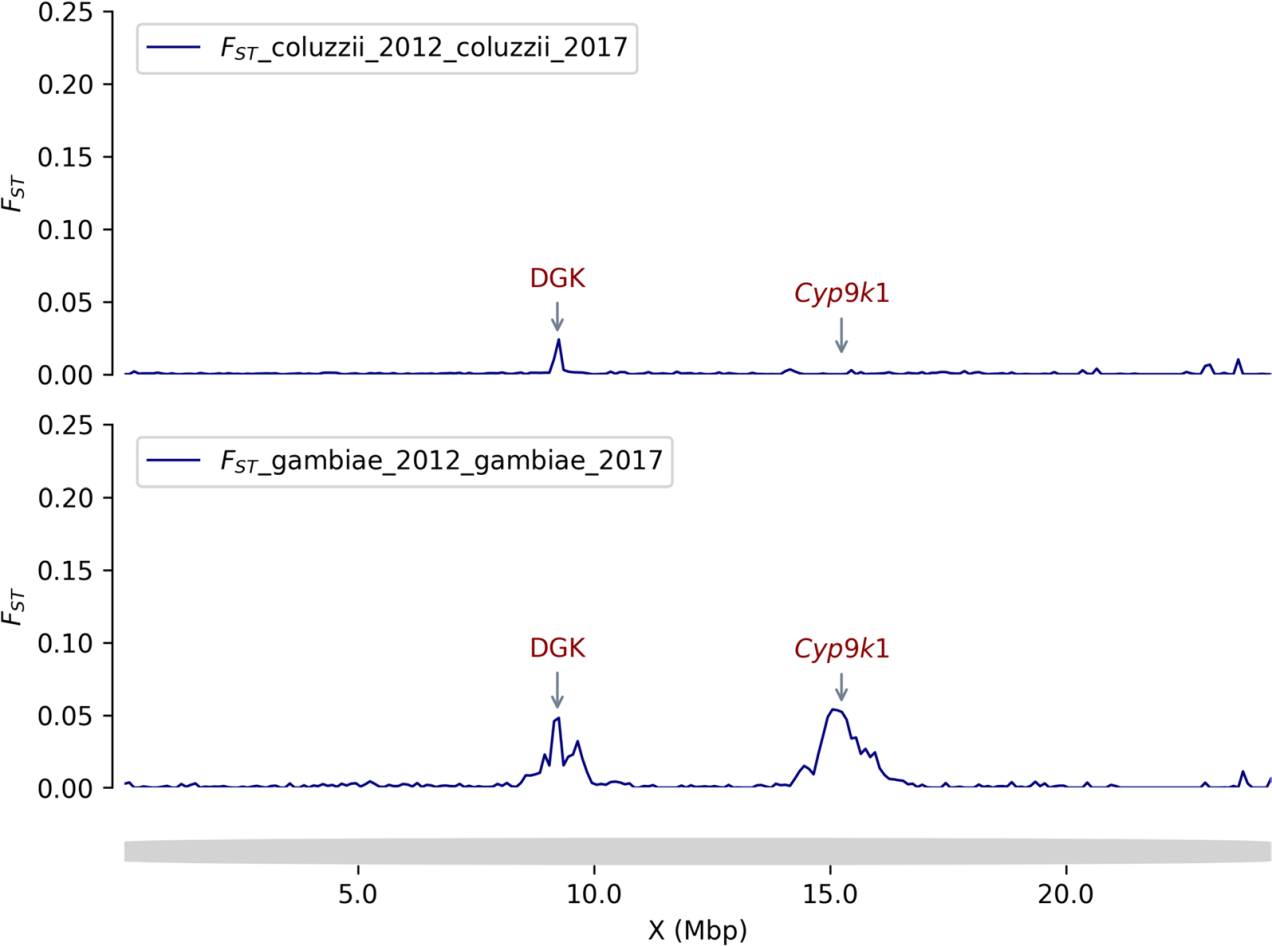
Genome-wide selection scan using genetic differentiation (*F*_*ST*_) across chromosome X (10 kb window) between populations of *An. gambiae* s.s. and *An. coluzzii* collected in 2012 and in 2017. The X-axis shows the positions of the chromosome X and the Y-axis shows the values of the 10 kb windowed *F*_*ST*_. Signals of positive selection (high genetic differentiation, *F*_*ST*_ > 0) were observed in the genomic regions corresponding to *Cyp9k1* and *DGK* genes within the *An. gambiae* s.s populations

The emergence of new forms of insecticide resistance may impact vector control strategy in unexpected ways. Recent studies reported new forms of resistance, especially behavioural changes (early and/or outdoor biting) in *An. gambiae* mosquitoes, in response to the intense use of LLINs causing residual malaria transmission [52, 53]. Here, a signal of positive selection was detected at the DGK locus and an increased frequency of CNVs of this gene within the *An. gambiae* s.s. populations (Figs. 8 and 9). No selection signal and no CNVs were detected in vector populations in 2012. However, in 2017, a signal of positive selection was detected in the DGK locus, with the presence of CNVs at increasing frequency (from 0.00 in 2012 to 16.67% in 2017) in vector populations (Figs. 7, 8 and 9). The evolutionary pattern of the DGK gene and its reported role in the adaptation of *Caenorhabditis elegans* and *Drosophila* spp. to environmental changes [54, 55], suggest that this gene may play an important role in the modulation of *An. gambiae* biology in response to the presence of vector control tools.

## Discussion

Significant advances have been made in the reduction of malaria burden using LLINs and IRS, but the efficacy of these tools is at risk due to the high adaptability of the vector populations to the anthropogenic ecological changes [42]. A better understanding of the molecular, ecological and evolutionary processes allowing vector populations to adapt to the environmental changes would help in the development of innovative tools to support the current control strategies in malaria elimination. This study generated whole genome sequencing data to investigate the genetic diversity, the population structure and the evolution of insecticide resistance variants in the *An. gambiae *s.l. populations in the western part of Burkina Faso.

### Population structure and vector control

*Anopheles gambiae *s.s. and *An. coluzzi*, originated from the previously described *An. gambiae* M and S forms have similar historical genomic and demographic backgrounds [36]. These results are consistent with this evidence and confirm the similar demographic history between these two species. The results also showed low *F*_*ST*_ between *An. coluzzii* and *An. gambiae* and are consistent with ongoing gene flow between *An. gambiae* and *An. coluzzii*. This supports previous studies in the same area showing strong evidence of gene flow between *An. gambiae* and *An. coluzzii* populations in west Africa [13]. In the context of genetic control, this lack of population structure between these species supports the ongoing hypothesis of the spread of gene drive constructs between *An. gambiae *s.s. and *An. coluzzii*, as previously observed with the insecticide resistance variants [38, 56]. The gene flow also contributes to the spread and the maintenance of genetic variants associated with insecticide resistance in vector populations [57].

### Bed nets—evidence and impacts of target-site and metabolic pyrethroid resistance

The insecticides pressure on malaria vectors lead to an emergence of new insecticide resistance variants that confer fitness benefits to vector populations [58]. This seems to be consistent and evident for the SNPs *vgsc-V402L* + *I1527T* which were identified at increasing frequencies in *An. coluzzii* populations. The rapid increase in their frequencies within *An. coluzzii* populations was expected as a recent study demonstrated the contribution of the *vgsc-V402L* to the pyrethroid resistance phenotype at a reduced fitness cost compared to *vgsc-L995F* [39]. The simultaneous emergence of different types of genetic variants in genes and gene clusters known to be involved in pyrethroid metabolism and target site mutations within the *An. gambiae s.l.* populations raises major concerns about the long-term efficacy of new pyrethroid insecticide-based control strategies such as LLINs.

Insecticide resistance has been widely studied in Africa, and recent works have reported multiple resistance variants spreading in vectors populations, jeopardizing NMCP efforts in control and elimination [18, 19, 59]. The implementation of LLINs incorporating piperonyl butoxide (PBO) aims to strengthen the efficacy of bed nets by inhibiting the cytochromes P450 enzymes responsible for the rapid detoxifying of pyrethroid insecticide [60]. High expression of the P450 enzymes was shown to increase the pyrethroid resistance and pre-exposure to PBO restores the susceptibility of malaria vector to pyrethroid [41]. In most cases, the main genetic variants responsible for the insecticide resistance in a given area remain unclear because of the high heterogeneity of the genetic markers mediating metabolic resistance. The results showed a high deletion rate of some CYP P450 genes in up to 80% of the vector populations. This situation could probably reduce the expression level of certain genes that are mostly targeted by PBO and increase the expression level of the least targeted one. However, the causes and the magnitude of the genes deletion and amplification in the mosquito genome remain unclear, but these variants do have a high importance in the rapid adaptation of vector populations to different ecological changes [16]. Moreover, the emergence of these additional new variants could enhance the level of pyrethroid resistance impacting the efficacy of the new vector control tools.

### Indoor residual spraying (IRS)—evidence and impacts of organophosphates resistance

Another major concern related to this multi-modal resistance is its threat on the effectiveness of the IRS. IRS is efficient when it is used in combination with LLINs because of its ability to target indoor resting pyrethroid resistant vectors [61]. Although the SNP *ace1-G280S* has long been identified in *An. gambiae* populations, its frequencies remained low in the populations probably due to its fitness cost in association with the kdr mutation [62]. In fact, the duplication of the *ace1* gene (*ACE1*^*D*^) seems to have completely solved the problem of genetic cost yielded by the SNP *ace1-G280S* [63]. Recent reports showed a wide distribution of this duplication in West African *An. coluzzii* and *An. gambiae *s.s. populations [19, 63] probably in response to the use of organophosphate and carbamates compounds in vector control. Similarly, the duplication of *Ace1* gene follows the same trend as the SNP *ace1-G280S* raising up in frequencies in *An. gambiae *s.s. populations. This clearly showed the close related link between these two variants in conferring high fitness to the mosquito populations. In addition, the CNVs identified at high frequencies in some of the *GSTe* and COE genes could increase the expression level of these genes. The simultaneous presence of these variants (CNVs and SNPs) in *ACE1*, *GSTe* and esterases genes within the same vector populations strongly contribute to increase the survival capacity of the vector against organophosphates and carbamates. This situation threatens the long-term efficacy of vector control interventions including the recently introduced pirimiphos-methyl-based IRS [19].

### Whole genome surveillance enables detection of potential novel insecticide resistance loci

Genome-wide scans for positive selection have proved their usefulness in the identification of genomic regions under divergent selection. In malaria vectors, under high insecticide mediated evolutionary pressure, these regions often have implications in insecticide resistance [64]. These analyses showed a signal of positive selection in a genomic region of the chromosome X corresponding to the DGK gene. Though there is currently no evidence of this gene’s association with insecticide resistant phenotypes in malaria vectors, studies in *Drosophila melanogaster* and *Caenorhabditis elegans* showed its potential involvement in the adaptation to environmental changings [54, 55]. In *D. melanogaster,* the *rdgA* gene, an orthologue of the *dgk* gene, is eye-specific, strongly involved in light signaling. Any genetic variant influencing the functioning of this gene would probably affect its sensitivity to light, as reported previously [54, 65]. Consequently, if light signaling is regulated the same way in mosquito species, the *dgk* gene may also be involved in the regulation of *An. gambiae* vision. In *An. gambiae*, a previous study showed a rhythmic expression of the genes involved in light signaling under the control of the circadian clock [66]. The expression pattern of these genes suggests their role in calibration of the mosquito visual system to start the nocturnal activities such as flight and biting. In response to the high coverage of LLINs, major changes were reported in vector biting behaviour particularly early biting and later biting activities in many areas of Africa [53, 67]. Even though no evidence was highlighted, these changes could be related to alterations in the expression of genes involved in the mosquito visual system.

Another possible implication of the *dgk* gene in the adaptation of vector populations is its indirect implication to insecticide resistance via the regulation of the acetylcholine release at synaptic junctions. In *C. elegans*, the *dgk* gene regulates the amount of acetylcholine in synaptic junctions and its inactivation causes a high susceptibility to aldicarb, a carbamate insecticide [55]. This report clearly showed the potential implication of the *dgk* gene in organophosphates and carbamates resistance *C. elegans*. If synaptic junctions are regulated in the same way in mosquitoes, any selective variant in the DGK gene would affect the acetylcholine release in synapses. These two hypotheses of the potential implication of the *dgk* gene in adaptations, either insecticide resistance or biting time change, were previously drawn up to explain the selection signal observed in the *dgk* gene [64]. These hypotheses appear to be different in both organisms, but the molecular mechanisms underlying the DGK gene function remain similar. Given this intriguing role of *dgk*, further studies are needed to deeply investigate in depth the role of the *dgk* gene (AGAP000519) in modulating malaria vector behaviour and its potential involvement in behavioural and genetic resistance. Therefore, future research would focus on understanding the genetic variation associated with dry season adaptation and population maintenance, as well as the molecular mechanisms underlying changes in vector behaviour.

## Conclusion

Investigation of genome variation in vector populations is crucial for understanding dynamics of populations in a given area and for guiding strategies of malaria control programs. This study showed a weak differentiation and strong genetic connection between *An. gambiae* and *An. coluzzii* populations in western Burkina Faso. The emergence of new variants together with the already existing pyrethroids resistance variants at high frequencies raise concerns about the long-term efficacy of new-generation nets impregnated with pyrethroids compounds. The long-term efficacy of pirimiphos-methyl-based IRS is also threatened because of the increasing frequency of the SNP *ace1-G280S* and the duplication of the *ace1* gene, which confer high fitness to vector populations carrying them. Genome-wide selection scans detected strong signals of positive selection in the genomic regions having an implication in insecticide resistance. This study highlighted the benefit of the molecular surveillance of malaria vectors for detection of new insecticide resistance variants, for monitoring the existing resistance variants and for getting insights in evolutionary processes underlying these variants.

## Supplementary Information

Supplementary Material 1: Fig. S1. Site frequency spectra in the 3L chromosome of the *An. gambiae* s.l. populations from 2012 to 2016 in the three villages). The X axis of each figure shows the minor allele frequency and the Y axis, the density of the variants. The colour boxes are indicating the sampling periods.

Supplementary Material 2: Fig. S2. PCA showing the year-to-yeargenetic structure of *An. gambiae* s.l. populations using the whole SNPs identified in the 3L chromosome. B. Year-to-year genetic structure of *An. coluzzii* populations; C. Year-to-year genetic structure of *An. gambiae* s.s. populations; D. Year-to-year genetic structure of *An. arabiensis* populations; These figures demonstrated the lack of geographic substructure within each species of the *An. gambiae* s.l. populations collected in different villages over the years.

Supplementary Material 3: Fig. S3. Genome-wide selection scan using genetic differentiationacross the genomeof *An. gambiae* s.s. and *An. coluzzii* collected in 2012 and in 2017. Signals of positive selection were observed in the genomic regions shown to be involved in insecticides resistance: *ace1*: acetylcholinesterase gene, *cyp6p*: Cytochrome P450 gene, *keap1*: Kelch-like ECH-associated protein 1, *vgsc*: voltage-gated sodium channel, *rdl*: resistance to dieldrin gene, *Dgk*: *Diaglycerol Kinasegene*, *cyp9k1*: Cytochrome P450 gene, *Mbp*: megabases.

Supplementary Material 4: Fig. S4. Heat map showing the CNVs frequenciesof the glutathione-s-transferase genes in the *An. gambiae* s.l. populations. The X axis shows the *An. gambiae* s.l. populations and the sampling periods. The Y axis shows the positions of the glutathione-s-transferase genes ID and the CNV type. The gradient colour bar shows the distribution of the allelic frequencies.

Supplementary Material 5: Fig. S5. Heat map showing the CNVs frequenciesof the carboxylesterase genes in the *An. gambiae* s.l. populations. The X axis shows the *An. gambiae* s.l. populations and the sampling periods. The Y axis shows the positions of the carboxylesterase genes ID and the CNV type. The gradient colour bar shows the distribution of the allelic frequencies.

Supplementary Material 6: Table S1. Non-synonymous SNPs frequencies of the *Vgsc* genewithin *An. gambiae* s.l. populations.

Supplementary Material 7: Table S2. Copy number variations of the cytochrome p450 genes and their frequencies within *An. gambiae* s.l. populations.

Supplementary Material 8: Table S3. Non-synonymous SNPs frequencies of the *ace1* genewithin *An. gambiae* s.l. populations.

Supplementary Material 9: Table S4. Copy number variations of the glutathione-s-transferase genes and their frequencies within *An. gambiae* s.l. populations.

Supplementary Material 10: Table S5. Copy number variations of the carboxylesterase genes and their frequencies within *An. gambiae* s.l. populations.

## Data Availability

Jupyter Notebooks and scripts to reproduce all the analyses, tables and figures are available in the GitHub repository: https://github.com/mkient/BF_Ag1000G_analyses. The SNPs and haplotypes data are available on the homepage of MalariaGEN and can be accessed using the malariagen_data package. The raw sequences in FASTQ format and the aligned sequences in BAM format were stored in the European Nucleotide Archive (ENA, Study Accession n° PRJEB42254).
