# Supplementary figures and images for: Whole-genome sequencing of major malaria vectors reveals the evolution of new insecticide resistance variants in a longitudinal study in Burkina Faso
Source: Malar J. 2024 Sep 17;23:280. doi: 10.1186/s12936-024-05106-7 (PMC11406867; doi:10.1186/s12936-024-05106-7)

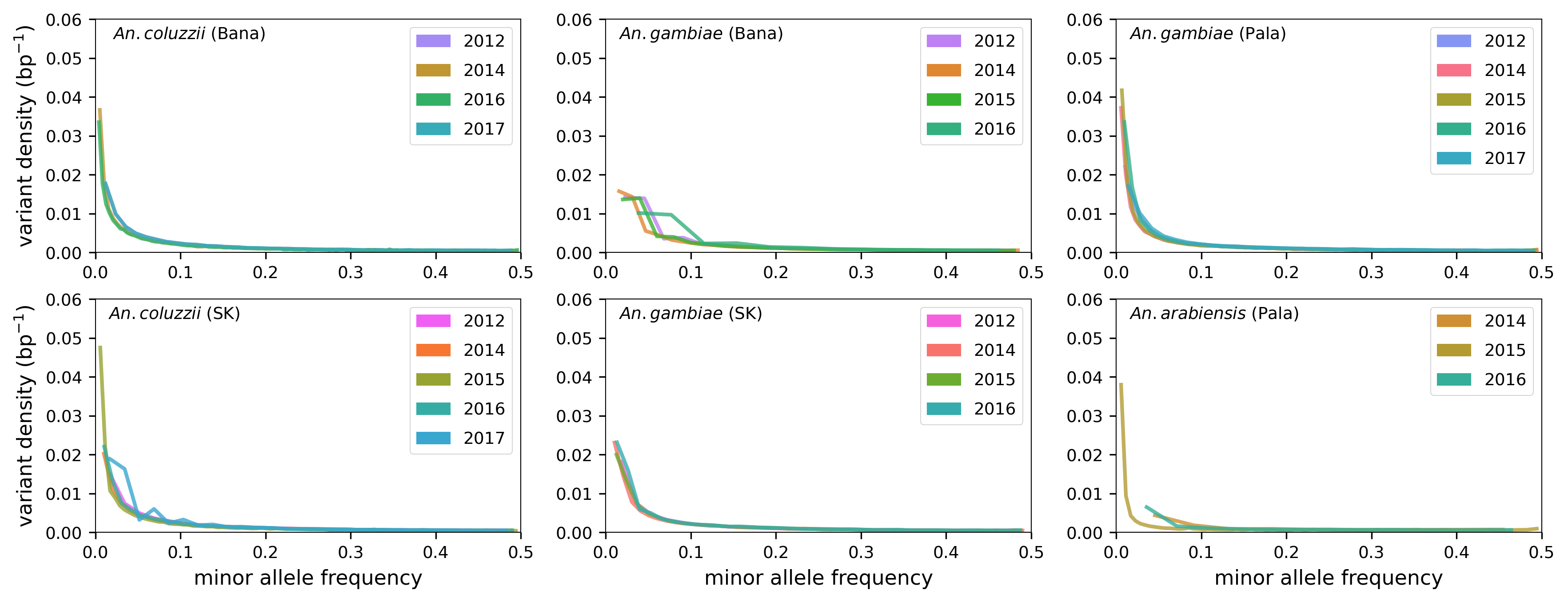

Supplement: Supplementary file 1 — Supplementary Material 1: Fig. S1. Site frequency spectra in the 3L chromosome of the An. gambiae s.l. populations from 2012 to 2016 in the three villages). The X axis of each figure shows the minor allele frequency and the Y axis, the density of the variants. The colour boxes are indicating the sampling periods. [file 12936_2024_5106_MOESM1_ESM.png]

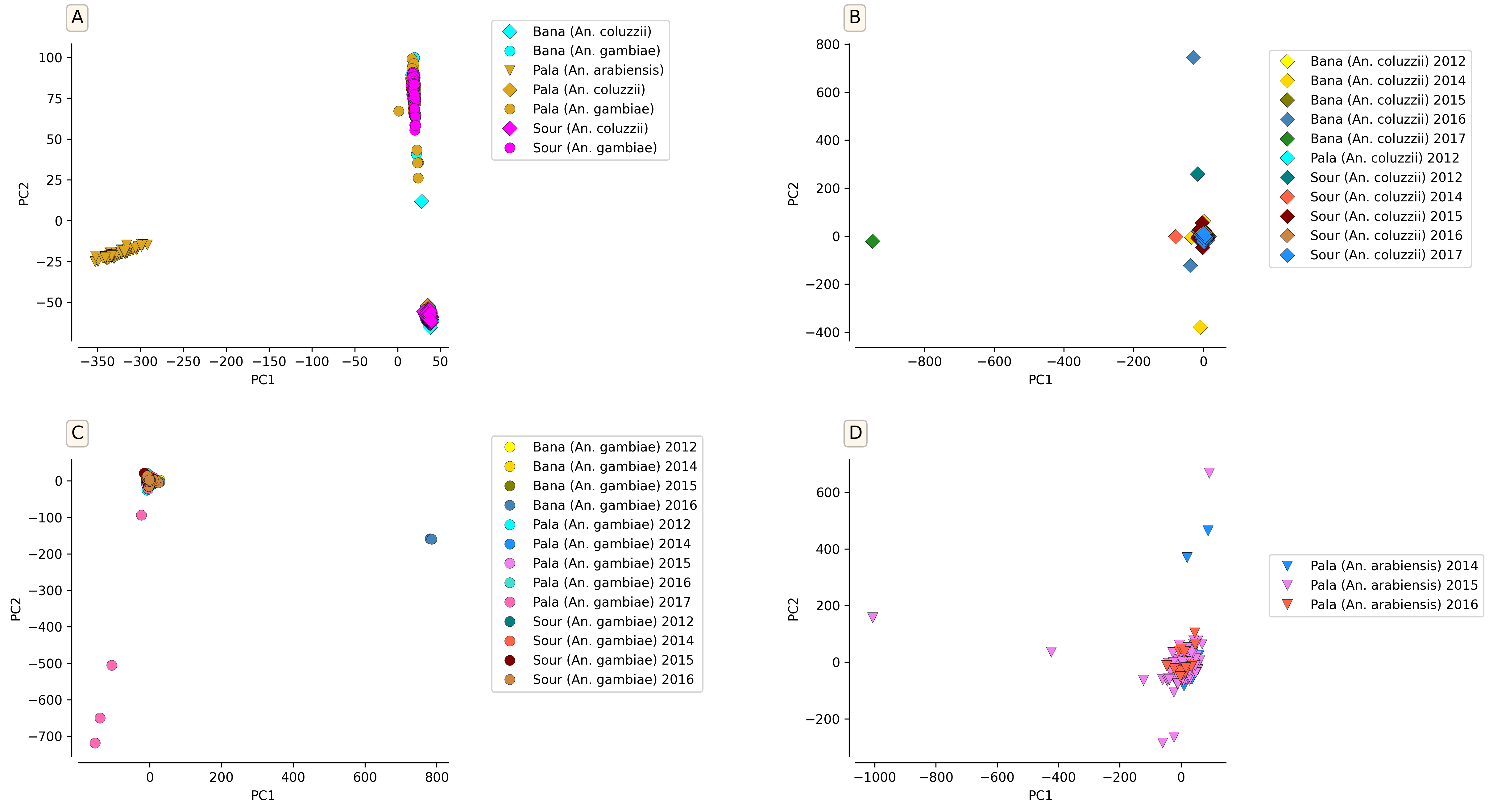

Supplement: Supplementary file 2 — Supplementary Material 2: Fig. S2. PCA showing the year-to-yeargenetic structure of An. gambiae s.l. populations using the whole SNPs identified in the 3L chromosome. B. Year-to-year genetic structure of An. coluzzii populations; C. Year-to-year genetic structure of An. gambiae s.s. populations; D. Year-to-year genetic structure of An. arabiensis populations; These figures demonstrated the lack of geographic substructure within each species of the An. gambiae s.l. populations collected in different villages over the years. [file 12936_2024_5106_MOESM2_ESM.png]

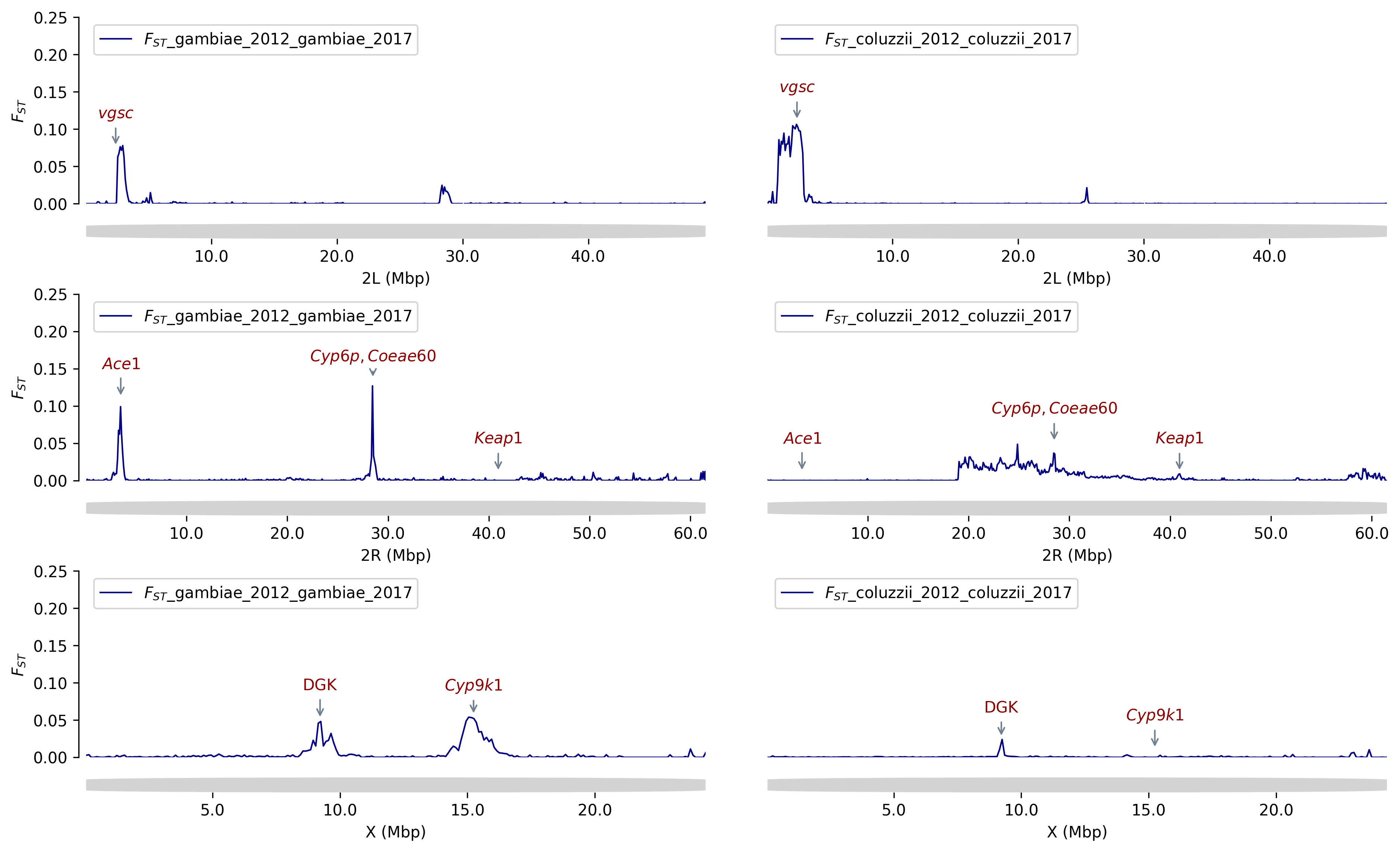

Supplement: Supplementary file 3 — Supplementary Material 3: Fig. S3. Genome-wide selection scan using genetic differentiationacross the genomeof An. gambiae s.s. and An. coluzzii collected in 2012 and in 2017. Signals of positive selection were observed in the genomic regions shown to be involved in insecticides resistance: ace1: acetylcholinesterase gene, cyp6p: Cytochrome P450 gene, keap1: Kelch-like ECH-associated protein 1, vgsc: voltage-gated sodium channel, rdl: resistance to dieldrin gene, Dgk: Diaglycerol Kinasegene, cyp9k1: Cytochrome P450 gene, Mbp: megabases. [file 12936_2024_5106_MOESM3_ESM.png]

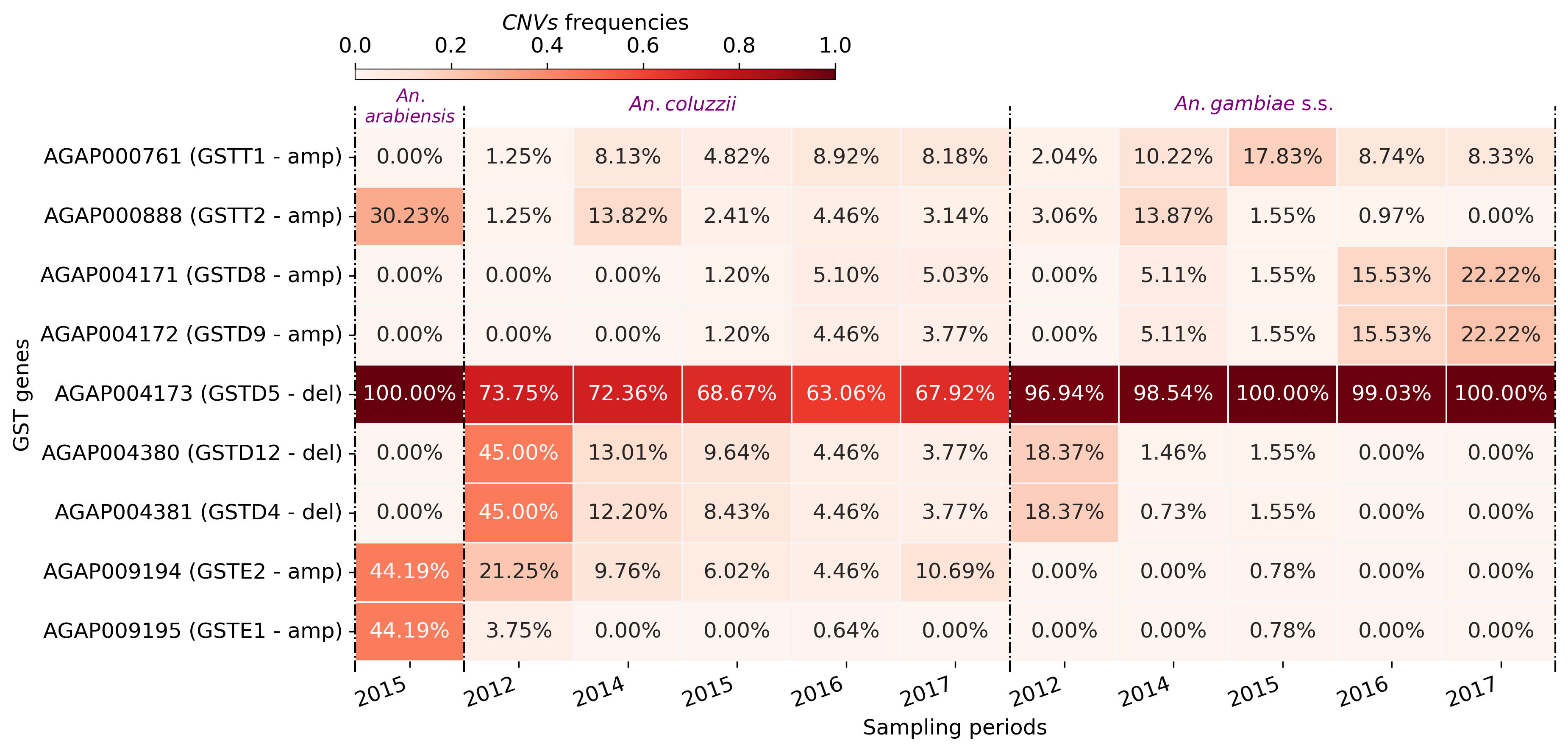

Supplement: Supplementary file 4 — Supplementary Material 4: Fig. S4. Heat map showing the CNVs frequenciesof the glutathione-s-transferase genes in the An. gambiae s.l. populations. The X axis shows the An. gambiae s.l. populations and the sampling periods. The Y axis shows the positions of the glutathione-s-transferase genes ID and the CNV type. The gradient colour bar shows the distribution of the allelic frequencies. [file 12936_2024_5106_MOESM4_ESM.png]

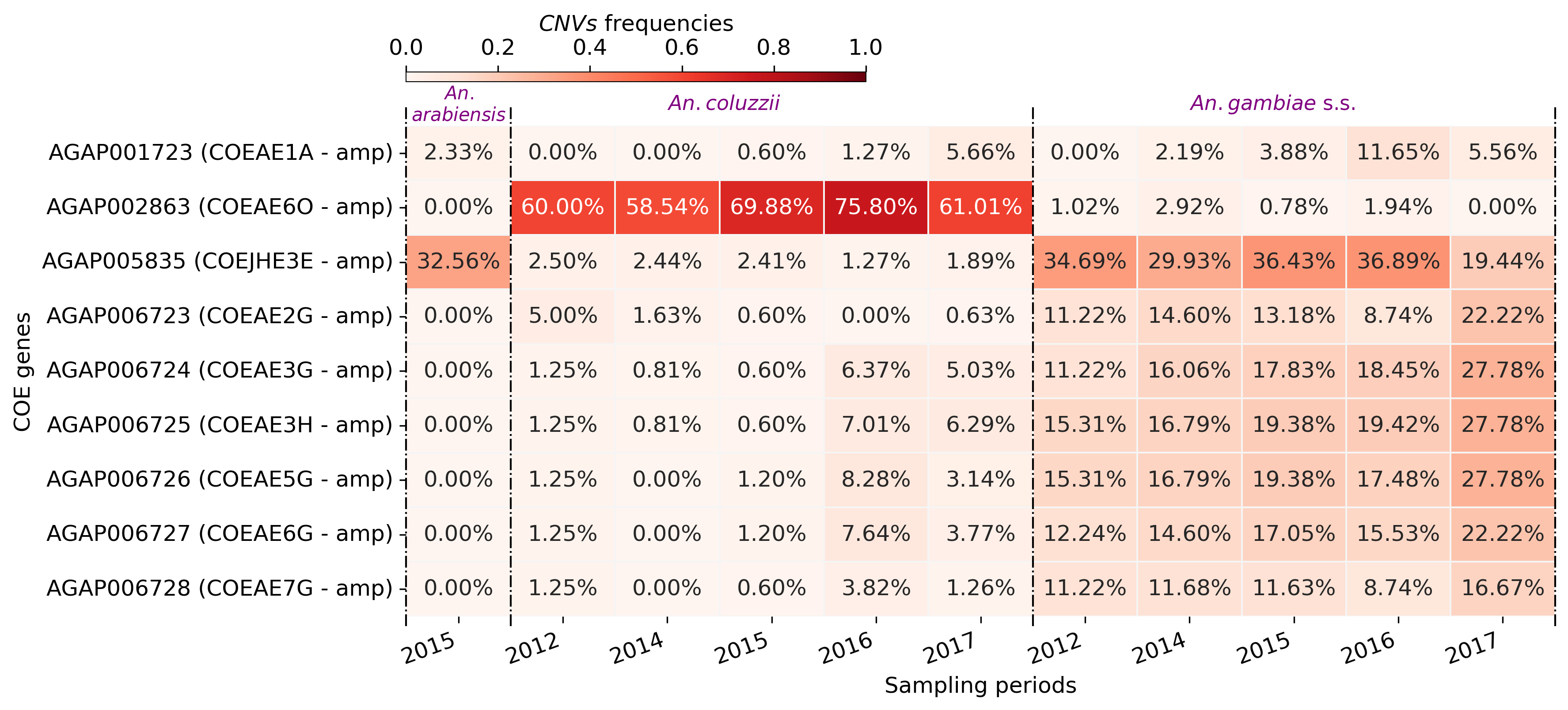

Supplement: Supplementary file 5 — Supplementary Material 5: Fig. S5. Heat map showing the CNVs frequenciesof the carboxylesterase genes in the An. gambiae s.l. populations. The X axis shows the An. gambiae s.l. populations and the sampling periods. The Y axis shows the positions of the carboxylesterase genes ID and the CNV type. The gradient colour bar shows the distribution of the allelic frequencies. [file 12936_2024_5106_MOESM5_ESM.png]
